# Climate, Fascism, and Ibex: Experiments in Using Population Dynamics Modeling as a Historiographical Tool

**DOI:** 10.1007/s10739-019-09579-0

**Published:** 2019-07-18

**Authors:** Wilko Graf von Hardenberg

**Affiliations:** Max Planck Institute for the History of Science, Boltzmannstr. 22, 14195 Berlin, Germany

**Keywords:** Fascism, Ibex, Population biology, Modeling, Nature conservation

## Abstract

In the interwar years the Gran Paradiso ibex population followed two subsequent, contrasting trends: a steady rise once the national park was established in 1922, followed by a precipitous fall after the Fascist regime took direct control of conservation in 1934, which almost led to the colony’s extinction. This paper addresses the issue of how models taken from population ecology may inform historical narratives. The data for the interwar years were analyzed using a statistical model based on climate and population density, which has proved reliable for most of the post-World War II period. The article highlights the pivotal role of anthropic variables in determining the inter-war trends and how these are best analyzed using historical scholarship.

## Introduction

Mathematical and statistical modeling has become a pivotal feature of population ecology in the decades since the field came to prominence in the 1920s through seminal work by people such as Vito Volterra and Alfred J. Lotka. After a difficult start, due to the limitations of early computational power, the idea of adopting mathematical methods to simplify ecological relationships and environmental interactions became gradually more appealing. Having initially been developed by scientists from disciplines outside ecology, mathematical representations of population trends had acquired a small but firm foothold in the field by the 1940s, and computer simulations became an essential tool in population dynamics from the 1960s onwards. Thus, theoretical, mathematically-informed ecology has become increasingly relevant, and as early as 1966, population ecology was considered the aspect of the discipline that was most influenced by mathematical thinking. In the following decades this approach developed hugely, in parallel with steady advances in statistics and dynamic systems theory and the downright explosion in the speed and accessibility of computation. Population models are now commonly considered among the most powerful and readily available tools to explain ecological processes through simplification and abstraction (Kingsland 1985, pp. 1–5, 106–116, 206–209; Israel 1988; Palladino 1991; Levins 1966, p. 421; Millán Gasca 1996, p. 347; Levin et al. 1997; Thieme 2003, pp. 1–4).

These models, which “however imperfectly, try to approximate the mechanisms of nature” (Cronon 1992, p. 1349), have been adopted by environmental historians over the years as tools to inform and improve their narrative efforts. In this vein, in this paper I borrow methods and tools from population biology in an attempt to see what role they can play in refining and clarifying historical narratives about nature conservation. Historians and ecologists, with all the differences in the methods by which they put their work into practice, share a common interest in answering questions historically (Kingsland 1985; Pooley 2013). Thus, as part of a broader debate on how to study socio-ecological systems, an increasingly rich literature has been deconstructing and analyzing the disciplinary interactions between ecology and history. Scholars have mostly paid attention to the contributions the latter can offer the former in gathering data and providing contextual insights that may help to better understand past ecosystems and ecology’s need for a thorough historicization (Pooley 2018; Higgs et al. 2014; Taylor 2013; Alagona et al. 2012; Szabó and Hédl 2011; Collins et al. 2010; Bowker 2000). However, rather than focusing on what the historian can do for the population biologist, in this paper I consider what the population biologist’s toolbox has to offer the historian.

**Fig. 1:**
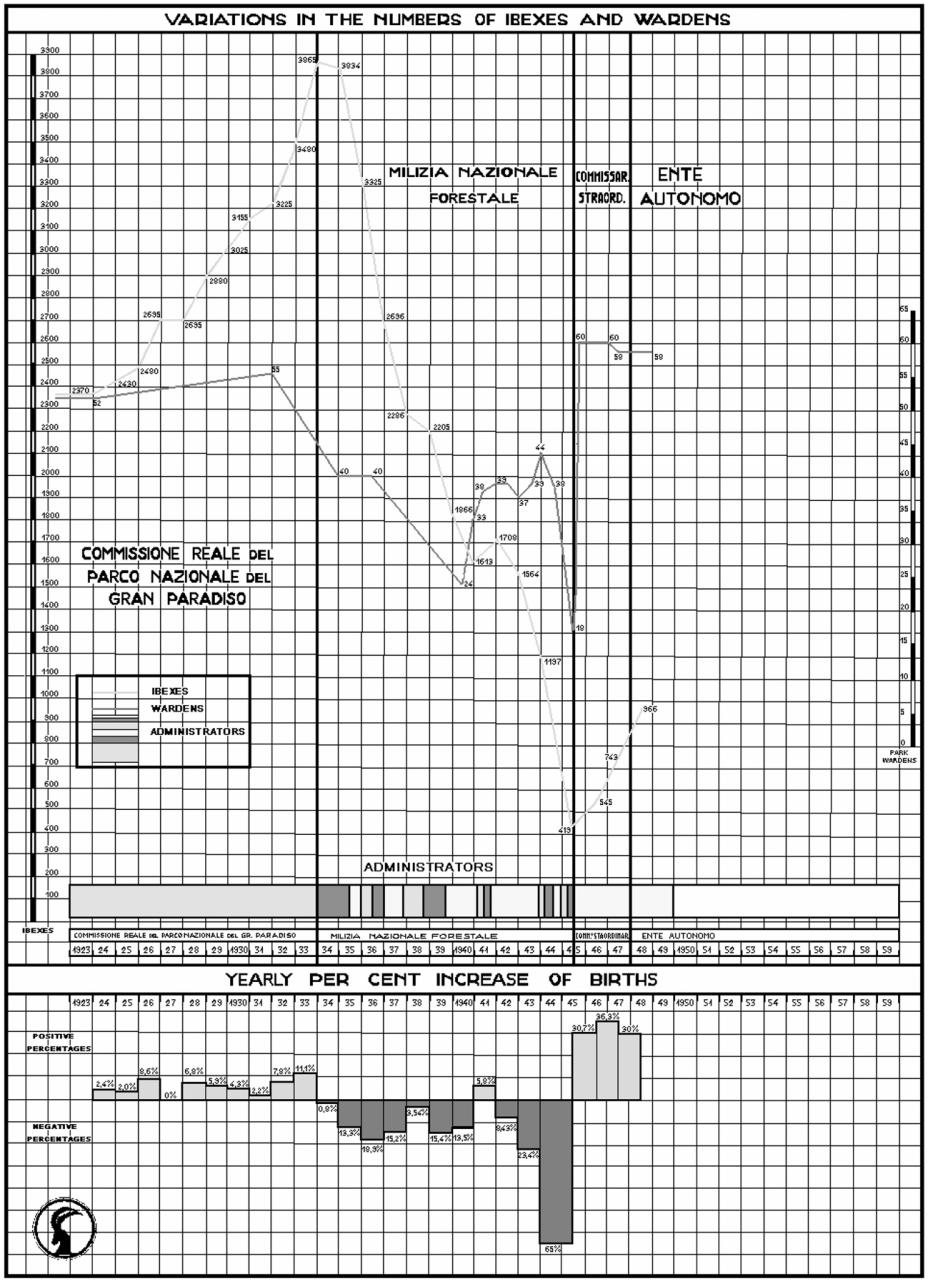
Variations in ibex population on the Gran Paradiso since 1922 Adapted by the author from Diagrammi (n.d.)

In particular, I explore whether, through modelling, it is possible to build a case for alternative explanations to the interwar dynamic of the ibex population of the Gran Paradiso National Park, Italy, which was marked by a steady increase until 1934 and then a steep fall in the following years (Fig. 1). This trend has customarily been credited to anthropogenic causes, such as the varying quality of surveillance and the impact of poaching. I discuss whether this dynamic could instead be explained by taking into account nothing more than ecological and climatic variables. In doing so, I explore, more generally, the potential of using biological modelling as a tool to clarify and discuss historical causation processes.

The area of the Gran Paradiso massif in northwestern Italy has historically hosted the last surviving colony of Alpine ibex (*Capra ibex*), from which, in a radical case of a “geographically discordant recovery pattern” (Estes et al. 1998, p. 473), all current populations in the Alps originate. Authorities were aware of the importance of conserving this remnant population since the early nineteenth century. In 1821, King Carlo Felice issued a total ban on hunting ibex and, in the 1850s, King Vittorio Emanuele II set up a royal hunting reserve in the region with its own dedicated corps of wardens whose mission was to preserve the endangered game. In 1919, as part of a wider attempt to divest himself of royal properties for economic reasons in the difficult years following World War I, his grandson King Vittorio Emanuele III donated the reserve to the state, with the caveat that it had to become a national park. This led, after some debate, to the establishment of the Gran Paradiso National Park in late 1922, just a couple of months after Mussolini’s Fascist movement ascended to power. Even though formally instituted by a coalition government led by the Fascist party, the park was essentially a product of political debates originating in the previous Liberal era. This was reflected in the autonomy granted to the park in its first decade of existence (Hardenberg 2010, pp. 142–147; 2014, p. 277; Passerin d’Entrèves 2000, pp. 81–87).

According to the annual ibex censuses, conservation in the park was initially a success. Various sources, including a graph that circulated in various versions in the aftermath of World War II and an internal memo from the 1940s, show that the number of ibex in the region increased from 2370 in 1922 to 3865 in 1933 (Diagrammi n.d.; Moser 1941; Barbieri 1947, p. 187; Pedrotti 2007, pp. 59–60). In 1933, Benito Mussolini’s government, as part of its move since 1925 towards the thorough centralization of the state and the implementation of totalitarianism at all levels, disbanded the park’s autonomous administration, steeped in the ideals of pre-Fascist liberal conservationism, and put the Fascist forestry corps *Milizia Nazionale Forestale* in charge (Aquarone 1965, pp. 46–47; Romanelli 1995, p. 156; Melis 2018; Hardenberg 2010, pp. 147–151). Under the direct rule of the *Milizia*, through the difficult years of World War II and its aftermath, the ibex population dwindled. In 1942 there were just 1564 ibex left in the park and, in 1945, only 419.

This sudden turn in the population trend in the years following 1933 has been customarily attributed to an increase in poaching due to the poor quality of surveillance provided by the *Milizia*’s unmotivated and inexperienced foresters, in contrast to that granted by the local wardens the park had hired in its first decade of existence (Videsott 1945; Berlanda et al. 1972). Anthropic variables have thus been at the core of the narrative of both the park’s successes and pitfalls: the life of a preserved species has been depicted as being completely dependent, for better or worse, on either the positive impact of conservation policies or the role of mismanagement, which enabled poaching.

In this article, I describe how the adoption of a model taken from the population biology toolset contributes towards understanding whether this was actually the case, or if more simple mechanisms were at play. To do so, I first present the original model (Jacobson et al. 2004), which has proven pretty reliable at predicting ibex dynamics in the Gran Paradiso National Park for the 1956–2000 period, using exclusively population and weather data. In the following two sections, I discuss the reliability of available animal censuses and weather data for the interwar years. Then I apply the model to these data to compare expected and historical trends, showing that it is not able to reproduce the steep decline the local ibex population experienced, according to field surveys, after 1933–1934 under the *Milizia* management. This is indicative of the fact that other factors besides weather variability and population density played a key role in determining the ibex population dynamic in the interwar years. Finally, I compare interwar ibex population trends on the Gran Paradiso under Fascist rule with those in the other Alpine national park hosting an ibex colony, the one in democratic Switzerland. This contributes towards highlighting the role of the historical and political contexts in explaining the causation factors behind population trends.

## The Post-World War II Model

In 2004 a team of climatologists and population biologists published a model of the dynamics of the ibex colony in the Gran Paradiso National Park, 1956–2000. Their model explains population trends as a function of the harshness of winter climate, population density, and interaction between the two (Jacobson et al. 2004). The model’s creators chose to use snow depth as the climatic variable in their model because it appeared more likely to directly affect ibex, even if good predictions could also be obtained with other indicators of winter precipitation. With deep, long-lasting snow cover, food becomes scarcer and the ibex require more energy for motion and nutrition. In addition, the risk of avalanches and consequent casualties grow in harsh winters. High population density increases intraspecific competition for limited resources. Furthermore, a severe winter climate generally intensifies the effects of density dependence.

Since the end of World War II, wardens in Gran Paradiso have conducted ibex population censuses twice a year. They follow established routes and search visually, with the aid of binoculars, for ibex within their habitat. The reliability of these surveys still needs to be evaluated in detail, but less documentary material is available to determine the quality of surveys the further one reaches into the past. Nonetheless, the study’s authors suggest “that the combination of experienced observers, fixed census routes and open terrain has provided population estimates with little or no bias” (Jacobson et al. 2004, Appendix A).

Jacobson et al. (2004) produced a series of simple stochastic models (Engen and Sæther 1998). Having estimated each model’s parameters fitting the first 20 years of data, which were made available by the park’s own Alpine Wildlife Research Center, these were used to validate the out-of-sample predictions with the actual population counts over the subsequent 20 years. This approach was especially innovative because it confirmed the models’ predictions against an extant series of long-term observations, rather than just describing the trend.

Here I focus on the two complete models, which use as variables respectively actual population density (D.1) and the logarithm of density (D.2) in the previous year, winter precipitation, and the product of the two. The inclusion of the product of population density and winter precipitation may seem redundant, but appears necessary to accurately model the interaction of the two and attempt to explain at least some of “the deterministic variability in ibex abundance” (Jacobson et al. 2004, p. 1604). The models have proven to be able to reproduce most of the postwar time-series, which they were explicitly developed to explain, including an unprecedented population increase in the 1980s and a harsh decline in the 1990s. They failed, however, to predict the apparent stability (or possibly stagnation) of the Alpine ibex population in low snow depth conditions after the mid-1990s. Researchers are still investigating additional explanatory factors for this. Mignatti et al. (2012) have, for instance, suggested new variables for consideration: the state of the pastures, parasitic infections, and interspecific competition with, in particular, chamois, as well as temporal variability in juvenile survival and the role of senescence.

Exogenous, anthropic factors—like those which have traditionally been deemed crucial for determining the peculiar trend of the interwar years as well as those that have often been excluded in population ecology models because of their uniqueness and non-repeatability—have already been suggested to explain the unexpected population trends of the 1990s (Lima and Berryman 2006; Pooley 2018, p. 4). However, while there is clear evidence of a radical change in the way the park was administered during the Fascist era, there is no similarly revolutionary event recorded for the late 1990s (Jacobson et al. 2006).^1^

## Assessing the Reliability of Animal Censuses

The main problems that arise in applying statistical methods to the long-term history of animal populations are the dearth of data, the limitations of their reliability, and the recurring need to look for viable proxies. Because of the varying relevance of certain facts in respect to others, different regimes and social structures produce different data. The historical method and its role in framing how and why data were collected in the first place has only been discussed to a very limited extent in the literature, an issue addressed extensively in a recent paper by Simon Pooley (2018, p. 2).

Gathering, collecting, and ordering data are always political acts. They are undertaken for specific aims and purposes that may differ radically from those of current practitioners and may be influenced by extra-scientific factors. Moreover, the quality of bureaucratic record-keeping and data preservation varies over time. The combination of these aspects greatly restricts our ability to compare datasets throughout history: once data produced by different regimes are merged for long-term comparison, they end up being “artifacts driven by present concerns” rather than internally coherent series (Pooley 2018, p. 3). Similar discourses can obviously be made about other kinds of historical data, such as economic and demographic series, but the problem seems particularly relevant in the field of population ecology because of the limited availability of continuous and reliable series of animal population data in historical times.

In this regard the case of the Gran Paradiso National Park, while definitely not unique, may be exceptional, as its long conservation history and the focus on just one iconic species has produced a relatively consistent and extensive data series. On the other hand, as is often the case with protected areas, it is not possible to apply commonly-adopted alternative methods to make assumptions about animal populations, such as estimating them on the basis of their ranges, since the censuses, if not the actual population, were limited to the park (Bonebrake et al. 2010, pp. 371–372). What also is lacking are data that have the degree of detail common in more recent surveys (e.g., detailed subdivisions by sex or age classes), which might allow more advanced and fine-grained models to be adopted. Unfortunately, additional data provided by a *Milizia* officer, who recorded a major decrease in birth rates and a worrying flattening of the sex ratio in the years immediately after the population decline began, cover too short a period or are too anecdotal to be effectively modelled (Moser 1941). Both might, however, hint towards additional ecological causes for the decline in the 1930s that reflect those suggested as further factors for the discrepancies between model and record in the 1990s (Mignatti et al. 2012). Historical data about other species in the region, such as the chamois, are unfortunately only recorded sketchily. Had they been available they could have helped to support possible narratives about interspecies competition with descriptive models.

A concept neatly expressed by Higgs et al. (2014, p. 499) in regard to historical interpretation can be repurposed here for biological modeling based on past data: it is, indeed, “always contingent on the kind of evidence available.” The problem is that, for the purpose of modeling, we cannot ask the data at hand to reveal any information they do not contain. Models are, in fact, extremely powerful tools, able to shed light on uncertain causal relationships. However, they require specific kinds of data and certain levels of reliability and precision that are not always available when using datasets produced in historically determined contexts.

A crucial point in this attempt to adopt population ecology methods to refine the narrative and to learn why the Gran Paradiso ibex population changed over time during the interwar years is to ascertain whether the animal censuses taken in those years are robust enough to be used for statistical analysis. Can the data gathered during the Fascist regime be accepted as a reliable account of the actual size of the animal population, or are there any clues that they might be somehow skewed? As by-products of specific regimes of fact production, animal censuses, both historical and current, may be biased in many ways: the motives for which they are taken, the methods and tools used to take them, and the training and expertise of those who take them may all affect the reliability of the data (Pooley 2018, pp. 2–3). Even uninterrupted series or extremely recent datasets may hide behind an appearance of stability major shifts in procedure, effectiveness, and observational techniques.

As noted above, very little documentation is available to support even the continuous reliability of the data used by Jacobson et al. (2004). Animal censuses are extremely labor-intensive, a fact that may further influence the trustworthiness of the count. For one thing, humans will necessarily have to go where the animals are, possibly disturbing them and thus distorting the results (Verma et al. 2016, pp. 75–76). As noted by anthropologist Crystal Biruk, “clean data—well-collected raw numbers—contain within them thousands of stories of their messy contexts of production” (Biruk 2018, p. 55). The details of such stories are often lost because of missing accounts. They can still be reconstructed, at least in part, by bearing in mind the broader social and political context at the time they were collected.

Various doubts may arise about the available interwar ibex censuses. To start, the way the wardens counted ibex before World War II was very different from the ground count method described above, which was only adopted after the war (Jachmann 2001). As the first post-World War II director Renzo Videsott reported, during the interwar years, instead of counting ibex following set routes, wardens would first attract animals to specific spots with salt and then count how many came there over three days in a row (Pedrotti 2014, p. 17). Geographer Giuseppe Barbieri remarked in 1947 that it was easy to imagine the lack of care with which the delicate counting operations were performed by the members of the *Milizia*, who were often sent to the Gran Paradiso as a form of punishment for discipline breaches. As he noted, “the least that could happen was that a group of ibex or chamois, moving, would be counted twice and another would not be counted at all” (Barbieri 1947, p. 187).^2^ A further question is whether the censuses reflect real numbers or the varying desire of different actors at different times to frame the *Milizia*’s conservation efforts, in either a good or a bad light, for political and propaganda reasons.

However, the fact that the same numbers appear in both a post-World War II record (Diagrammi n.d.)—when the park’s new administrators were striving to outline the fallacies, faults, and shortcomings of the Fascist *Milizia*—and in a report written in the 1940s by one of the officers of just the corps that managed the park after 1933, major Moser (1941), seems to support the consistency of the data. Even should we suspect that the census numbers may be biased for some other reason, the severity of the post-1933 population decrease appears to be confirmed since this is supported by documents produced under radically different data production regimes.

On the other hand, one could think that the pre-1933 data, which had already been published in a 1935 article written by another *Milizia* officer (Verger 1935), are an exaggerated representation of the real trend, produced for reasons of Fascist propaganda. However, the fact that the same data were also used in the post-World War II account, and that Italian conservationists always praised the park’s autonomous administration’s good conservation practices prior to 1933, may imply that, again, they are at least accurate in giving an idea of the trend (Diagrammi n.d.; Peretti 1932; Festa 1933).

Another bias that could affect data gathered in the Fascist era censuses is linked to the relationship between the number of wardens and the number of ibex counted. It is possible that the reduced number of wardens after the *Milizia* took over full responsibility for the park did not actually lead to the presumed worsening of surveillance, subsequent increase in poaching rates, and, thus, an actual reduction in stock. It might instead have caused a less effective census methodology or a lack of data for some areas, leading to fewer animals being counted (or estimated) than those that were actually there. Without any independent sources to consult, it is almost impossible to determine which way the causal relationship went. It must, however, be recorded that, while the trend in their numbers was indeed declining, the wardens dipped below 30 (see Fig. 1)—the number employed by the modern surveys used by Jacobson et al. (2004, Appendix A)—only twice, and both times only very briefly. Thus, the presence of about 40 park wardens throughout most of the 1930s should have granted the theoretical possibility of performing censuses over roughly the same territorial extent as before 1934 and after 1956.

As regards the matter of the new wardens’ lack of expertise and local know-how as compared to those hired by the park administration before 1933, mentioned also by Barbieri (1947), Moser’s insider report (1941) makes me lean towards an actual reduction of stock rather than a mere underestimation. For one thing, *Milizia* wardens were still accompanied by expert local auxiliary wardens with direct knowledge of the territory until 1941. On the other hand, it seems reasonable to assume that, as an officer of the *Milizia*, Moser would have preferred to criticize the wardens he commanded for their ineffectiveness in doing a survey once a year, had this been the case, rather than claiming they were bad at their surveillance job all the time. Hence, I feel confident in asserting that the interwar animal censuses accurately represent the ibex population trend (if not their exact numbers). This stands true even if, as Videsott claims, censuses in the interwar period were taken in May, while Jacobson et al. used the autumn counts in their computations (Pedrotti 2014, p. 17; Jacobson et al. 2004, p. 1599). In fact, according to von Hardenberg et al. (2000), spring and autumn figures are statistically strongly correlated, allowing either to be used in modelling, as both provide a fairly accurate record of the population dynamic.

## Identifying Usable Weather Data

A further necessity encountered in adopting Jacobson et al.’s (2004) model as a tool to analyze ibex population dynamics under Fascist rule was to find a continuous and reliable series of weather data for the period. Even though the national park administration had always actively promoted research on the area’s weather conditions and installed fifteen rain and snow gauges throughout the region, there are no usable data on snow depth or temperature in the Graian Alps for the interwar years. Observations were repeatedly interrupted by material failures in the instruments, which proved to be unsuitable for the high mountains’ cold climate (1929 Minutes). The available data, collected by the Ministry of Public Works’ hydrographic service, are very limited in quality and extent, providing snow depth just as monthly averages at first and then at ten-day intervals. The longest available continuous series ends in 1939 and was collected at a much lower elevation than the series analyzed by Jacobson et al. (2004). The only accessible data are precipitation measurements taken from a number of meteorological stations in and around the Gran Paradiso National Park. It was, thus, necessary to test the model using only winter precipitation instead of snow depth. In this context, winter is defined as the period from November to April, following Jacobson et al. (2004) and taking the specific climatic conditions of a high mountain region into account.^3^

Total precipitation is a good proxy for snow. Jacobson et al. (2004) have shown that winter precipitation and snow depth data from the meteorological station at Serrù dam in the postwar years are both significantly correlated with changes in the total ibex population. It is worth mentioning that it is still difficult to capture accurate measurements of the partitioning between liquid and solid precipitation nowadays due to a number of issues, including the unpredictable effects of wind. It is assumed here—since we are discussing events occurring on a historical scale, not apparently subject to major changes in the way climatic variables interact—that the same correlation Jacobson et al. (2004) detected for the post-World War II era is valid for the interwar years. Even assuming that anthropogenic climate change may have radically changed precipitation trends in recent decades, there is no reason to think that this would have affected the correlation between high altitude precipitation and snow depth almost a century ago. Furthermore, if higher temperatures in recent years may be causing precipitation to manifest as rain more frequently than snow, then the trajectory of the historical development of global warming means that temperatures would have been lower in the past, actually increasing the likelihood that precipitation at high altitudes would have appeared as snow (Rasmussen et al. 2011). According to Cassardo and Badino in their overview of historical climate change in Valle d’Aosta, snowfall was relatively steady in the region throughout the twentieth century up to 1982, with years having more or less snow alternating fairly regularly. Snowfall was relatively high across the whole region between 1935 and 1937 and, according to anecdotal evidence, winter 1935–1936 was particularly dreary, with possible negative impacts on the ibex (Cassardo and Badino 2006, p. 103; Cordier-Goni 1937, p. 219).

**Fig. 2:**
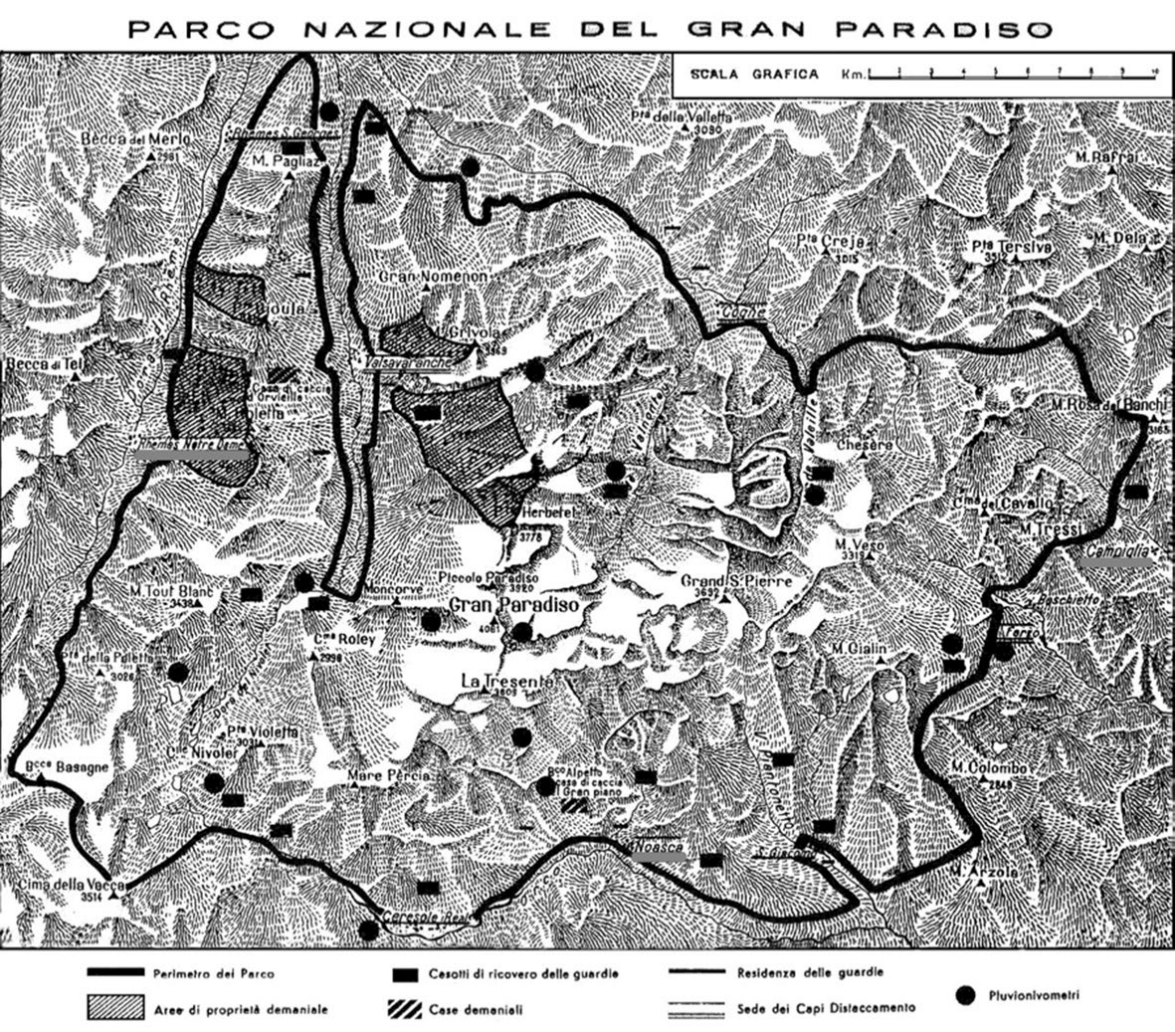
Selected stations (Rhêmes Notre Dame, Noasca, and Campiglia) underlined in grey. Image modified by the author, original taken from Commissione Reale del Parco Nazionale del Gran Paradiso (1925)

Out of all the available meteorological stations, I have chosen to use the three with the most complete datasets. These stations are located at the three corners of the park, thus giving a view of the park’s overall climatic conditions, overcoming valley-specific microclimates (Fig. 2). However, these three meteorological stations are located at lower altitudes than the one at Serrù dam that Jacobson et al. used in their model (2004, p. 1599). No continuous data from higher altitudes is available for the interwar years, both because Italy did not have a working network of high-mountain meteorological stations at that time and because the ones set up by the park administration continued to break down (Vanni 1950; 1929 Minutes). Even though this might affect the total registered amounts of rain and snow, it is less likely to affect inter-annual variations and trends in precipitation, which are associated with changes in large-scale circulation patterns. Moreover, the correlation between average precipitations in the three stations examined in this article and the average seasonal snow depth at the Rhêmes-Notre-Dame station (at the highest altitude of all three) in the period for which both data series are available (1922–1938) is fairly good, with an R^2^ of about 0.68.

**Fig. 3:**
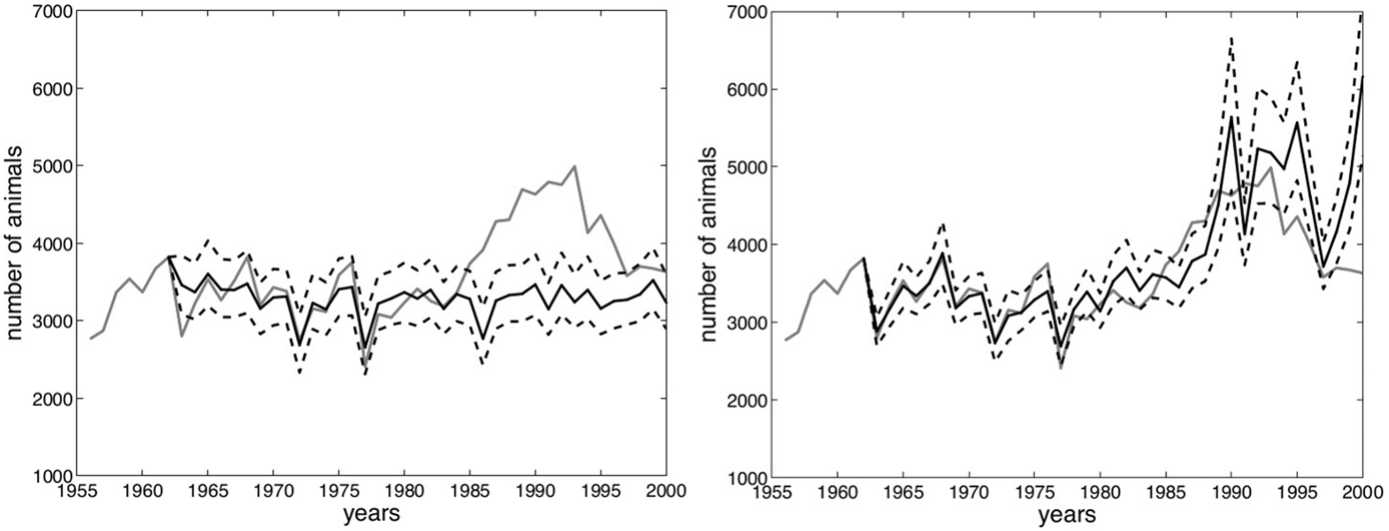
Jacobson et al.’s D.2 model (2004) using rain from the three new stations (left) versus the same model using snow depths at Serrù dam (right). The gray line denotes the registered development of the ibex population on the Gran Paradiso massif, while the continuous black line represents the average of 1000 stochastic model realizations. The dashed lines represent the bands within which 90% of the group of stochastic model realizations fall

Running Jacobson et al.’s D.2 model (2004, pp. 1605–1607) with the winter precipitation recorded at the three selected meteorological stations after World War II, rather than the snow depth data from Serrù dam employed by Jacobson et al. (2004), produced reliable predictions (Fig. 3). Also, testing their D.1 model with data from the three selected meteorological stations produced viable results for both before and after World War II. However, these precipitation data do not provide any information about the permanence of snow on the ground or on snow depth. Therefore, with only the precipitation data, the model can apparently not reproduce the post-1980 peak, which—as Jacobson et al. (2004) affirm—was heavily influenced by the shorter permanence of snow on ground and by lower snow depths. Nonetheless, the model seems able to reproduce a long-term stability of the ibex population somewhere between 3000 and 4000 units, which reflects the outcomes obtained using the snow depth data from Serrù dam. The inability to model extreme trends without data for snow depth might also be the case for the years after 1933 but, as the next section shows, there is an abundance of clues in documental sources to support the theory that the steady fall in the ibex population was indeed due to political and social variables that are difficult, if not impossible, to model solely on the basis of ecological feedback (Collins et al. 2010, p. 356).

## Comparing Population Trends

I have run Jacobson et al.’s (2004) model on the 1923–1942 period using the data described in the previous two sections. I have chosen to end the period under analysis in 1942 instead of continuing until the final years of World War II, when the conflict raged in the valleys of the Gran Paradiso National Park. This is when the rain data series collected in the selected meteorological stations was interrupted by the outbreak of civil war. This choice also helps to normalize some potential data inaccuracies and skews, such as the lack of an ibex population data point for 1944. It also avoids the need to consider the huge decrease in ibex stock between 1943 and 1945, which was almost certainly caused by warfare and the difficult conditions of the region at the end and in the immediate aftermath of the conflict, rather than by the quality of surveillance offered by the *Milizia* (Videsott 1945, pp. 24–25).

The real challenge I faced in adapting Jacobson et al.’s 2004 model to the interwar ibex population dynamic is that the lack of fluctuations in the population during the 1920s and 1930s makes it difficult to apply it as it stands. In fact, in contrast with the 1962–1980 period, when the total ibex population ranged between about 2600 and 4000 with “no visually apparent trend,” the census for the Fascist era seems to be split into two easily recognizable general trends: an exponential increase until about 1934 and an almost uninterrupted decrease afterwards. When applying the model to the Fascist years, one risks only detecting the exponential growth without explaining the slight annual variations in stock. Thus, the model unfortunately has only limited reliability in explaining the ibex population trend during the Fascist era (Jacobson et al. 2004, p. 1601; Provenzale to author 2009).

**Fig. 4:**
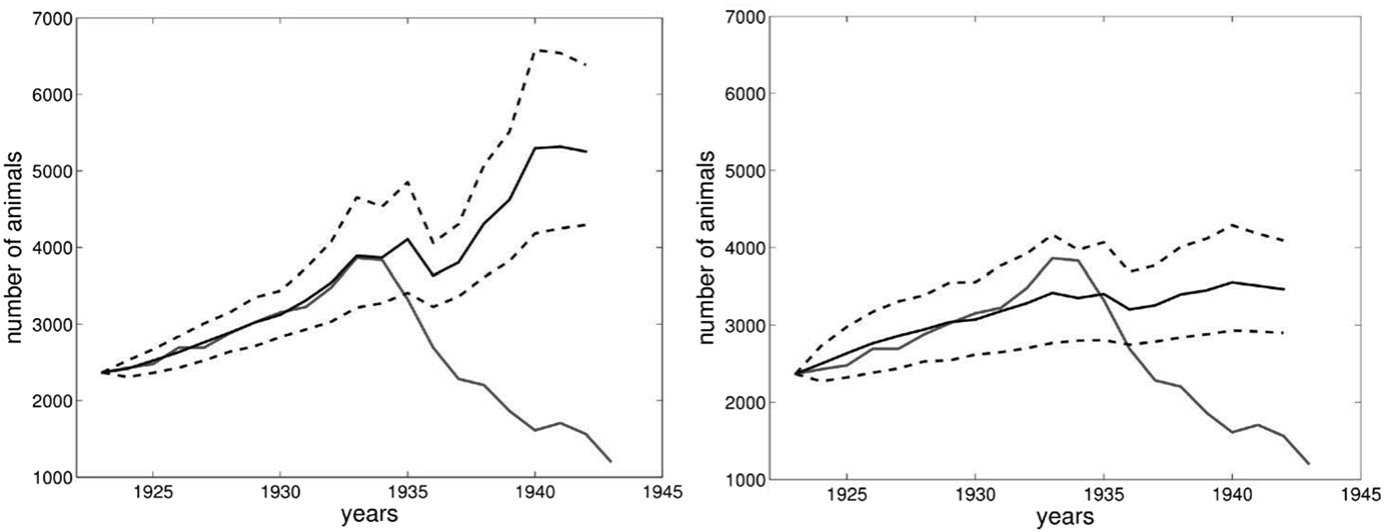
Model fitted to 1932 (left) and 1934 (right). See Fig. 3 for the meaning of the different lines

By fitting the model to the data in my sample until 1932, it is possible to reconstruct the light fall of 1933–1934 using only population density and precipitation. If we increase the number of years to which we apply the model, also including 1933 and 1934, we do not gain any additional explanation for the subsequent population decline but rather worsen the model’s predictive power. Considering that, despite all its limitations, the model is still able to reproduce the slight population decrease of 1934, it may be assumed that the latter was produced by natural causes related to population density and snow depth (Fig. 4).

As mentioned in the previous section, anecdotal evidence and reconstructions of the region’s historical weather show that the winters were particularly harsh from 1935 to 1937 (Cassardo and Badino 2006, p. 103; Cordier-Goni 1937, p. 219). This was not anomalous: high snowfall trends also occurred in 1975–1982. Then, however, the ibex population did not undergo a continued trend of decrease like that experienced during the Fascist era. Moreover, these weather trends do not lead the model to replicate the population slump registered in the surveys. It thus becomes evident that some other factor must have been responsible for the collapse of the ibex population after the—probably climate-induced—crisis of 1934. On the basis of the available historical documentation, I believe that this further decline must, indeed, have been related to a worsening in surveillance and an increase in poaching during the *Milizia’s* administration, in full accordance with the customary narrative about the Gran Paradiso National Park, also evidenced by Moser’s cited internal memo (1941). Moser was a rare example of a *Milizia* officer whose professionalism and dedication to conservation was recognized by his political opponents: in later years he would develop a frank, if conflictual, relationship with the main actor in the post-war re-establishment of an autonomous park administration, Renzo Videsott (Piccioni 2010, p. 142).

Moser stressed that the ibex population had started to decline steadily from 1935 onwards, just after the *Milizia* had taken over control of the park, and suggested the need to re-establish some form of autonomous administration, or at least to ensure that the commanding officer could focus exclusively on park business during the hunting season, from June to November, and be appointed to longer terms of office. Since the *Milizia* had taken over, commanding officers had, in fact, been rotated quite often, with negative impacts on the continuity of service (Fig. 1). Moser excluded exogenous factors, such as epidemics and avalanches, from the possible causes of the decline. He believed that, besides the migration of about 200 animals towards a neighboring hunting reserve due to the disturbances provoked in 1940 by the war on the French front, the main cause was an increase in poaching incidents after 1935, a direct consequence of the reduction in the number of wardens and of the steady decline of the region’s economic conditions.

One could argue however that since—using post-World War II precipitation data from the three selected meteorological stations—Jacobson’s model fails to predict the unexpected population increase of the 1980s, it is doubtful if it would be able to do so for the decline of the 1930s. In reality the results are not this easily comparable: in fact, using precipitation in the three selected stations, the model redraws the trend for the 1980s and 1990s as one of long-term stability, rather than one of boom and decline (see Fig. 3), while, applying it to a sample until 1932, it models a steady and continued increase for the 1930s, a trend that is diametrically opposed to that which was historically recorded (see Fig. 4). It must also be noted that this prediction of continued increase is given under climate conditions that appear way more extreme (and thus, in theory, inducive of a population decline) in the 1930s than in the 1980s (see Fig. 7).

**Fig. 5:**
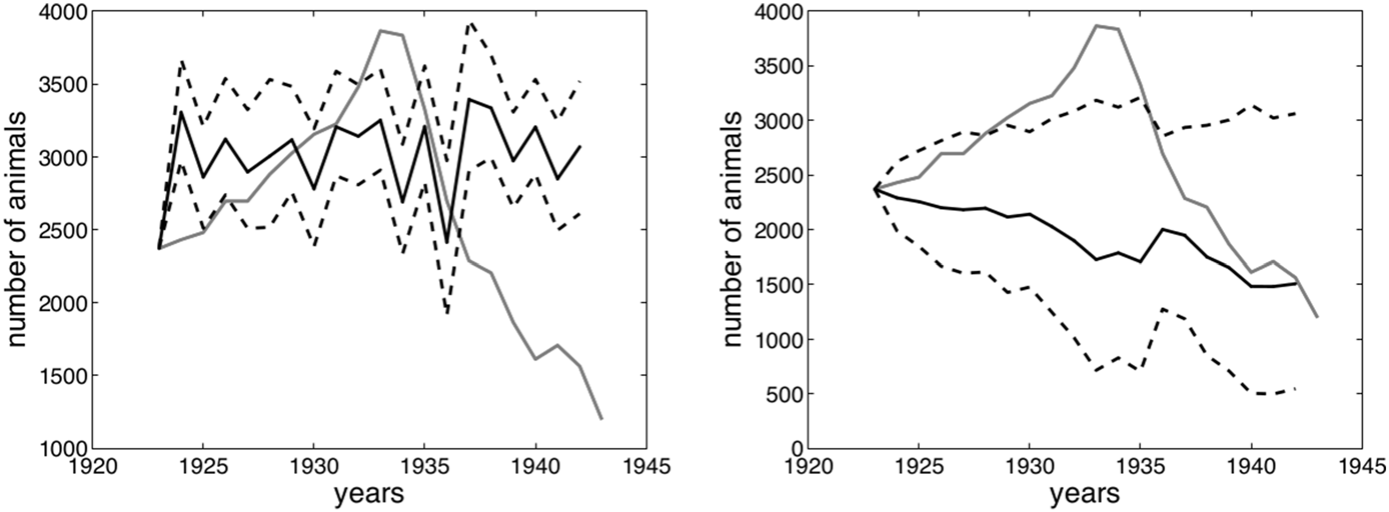
Model fitted to post-World War II data (left) and to data from 1923 until 1941 (right). See Fig. 3 for the meaning of the different lines

I have also tried to apply the model to post-World War II data and run it through the interwar years, and then to fit the model to a sample of all the data available until 1941. In both cases, as Fig. 5 shows, the model did a very poor job of reproducing the actual interwar population trend, further supporting the idea that it is not possible to replicate the Fascist era data series using this kind of model exclusively. The factors available to explain the ibex population trend between the two world wars, which reflect the customary narrative adopted after the end of the Fascist regime are thus: a relatively low, but still substantial, population just after World War I, a steady population growth during the first years of the Gran Paradiso National Park from 1922, a change in the way the park was managed in 1934, a relatively sudden decrease in the number of wardens, and then a continued decrease in the total ibex population.

If we look, for instance, at the trend in the number of park wardens, we can visually establish (see Fig. 1) how major changes in the ibex population usually happened with a 1-year lag in respect to the variations in personnel. The decrease in the number of ibex in the course of 1935 followed the dismissal of 16 wardens in 1934, and the slight trend reversal in 1941 was preceded by the employment of nine new wardens just a few months after their number had plummeted to just 24. Similarly, the great variability in the number of wardens that characterized the age in which the *Milizia* managed the park highlights how stability in their numbers during the 1920s effectively contributed to a steady increase in the number of ibex.

**Fig. 6:**
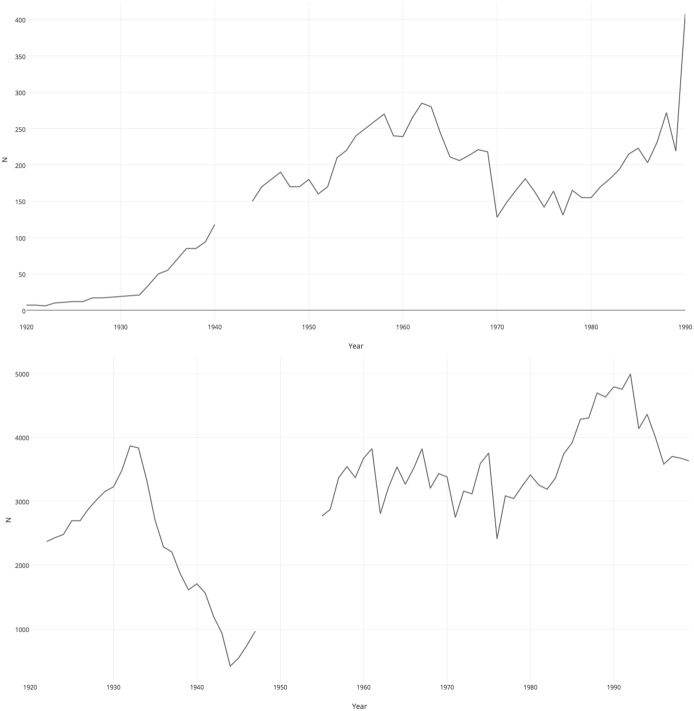
Ibex population trends throughout the twentieth century in the Swiss National Park (above) and the Gran Paradiso National Park (below)

As a means of comparison that may contribute towards clarifying causation, I have also examined the relationship between ibex stock and precipitation trends in the Swiss National Park that was established in 1914, a prime example of so-called ‘total preservation’ (Kupper 2014). Ibex had disappeared from this area in the early modern era, but were reintroduced in the early 1920s with cubs smuggled from the Gran Paradiso (Bundi 2006; Schneider 2006). The Swiss park’s first 20 years were marked by a steady growth in ibex population—even swifter than in the Italian case—which does not seem to have been influenced in any way by climatic variables. During the interwar years, the ibex population in the Swiss National Park was clearly in a phase of exponential growth, as is typical for founder populations, because of an extremely low initial density, well below the region’s carrying capacity (Fig. 6). The continued low population density overcame any other variable in determining population trends. Nonetheless, comparing graphically the long-term precipitation and population trends in the two parks provides some interesting supporting evidence for the need for an anthropic, or even eminently fascist, explanation for the sudden drop in the number of ibex in the Gran Paradiso after 1934.

**Fig. 7:**
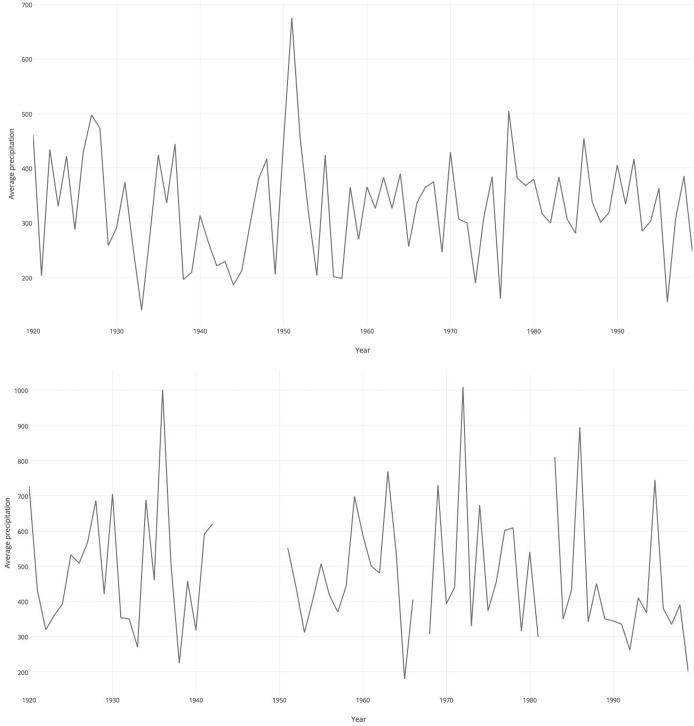
Winter precipitation at Buffalora station near the Swiss National Park (above) and the average precipitation in the three stations in the Gran Paradiso area discussed in this article (below)

The precipitation trends for the winter season follow, as expected, similar patterns of great variance over the whole twentieth century (Fig. 7). So do the overall population patterns in the post-World War II period. Even without testing the model on the Swiss data, it seems plausible that population density and snow depth (or precipitation as a proxy) played a role in determining ibex population trends there as well. What is missing in the Swiss National Park is any unexpected population decline at a time of relatively stable climatic conditions. This comparison does not provide definite proof, but is a further clue that something must have gone awry in the Gran Paradiso National Park’s wildlife management practices after 1934.^4^

## Conclusions

In this paper I have combined tools taken from recent developments in population biology and traditional historical methods to analyze animal population trends in Italy’s Gran Paradiso National Park during the interwar years. While the statistical model was not able to reproduce population dynamics beyond 1934, its adoption has allowed me to refine and strengthen the customary narrative blaming the Fascist regime and its policy choices for the decline in the number of ibex in the area between 1934 and 1943. Beyond this, the study also serves a broader purpose, presenting a case for the usefulness of statistical modeling for historians interested in assessing and disentangling the combined causal impact of environmental and anthropic variables.

Considering the noted limitations of the available data and the major difficulties in numerically representing a vast number of variables, even rough models may indeed turn into extremely useful tools for historians, enabling them to check theories and validate trends. For instance, in the case analyzed in this article, the fact that the observed interwar ibex population trends do not match those predicted by the model supports the idea that historians’ critical tools and knowledge around political and social changes may contribute towards identifying variables that have not been—or could not be—modelled, providing more holistic explanations for radical and sudden changes in the trends (Jørgensen 2014, p. 484; Scott 1998, pp. 30–32; Levins 1966, p. 421; Orzack and Sober 1993, pp. 534–535).

Partly because of the lack of certain kinds of data, Jacobson et al.’s (2004) model could not produce an alternative narrative explaining population trends and decoupling them from the (in)effectiveness of human conservation efforts before World War II. Applying the model to the interwar data, however, contributed to strengthening the customary narrative by confirming that the decline occurred mainly, if not exclusively, due to the worsening of conservation practices after the *Milizia* started managing the park. The model is able, up to a certain point, to explain trends with an extremely simple and elegant formula. As shown, such explanations may also be buttressed through comparison by looking at the relationship between animal population trends and socio-political settings in other contexts.

Statistical modeling also helps historians to avoid overthinking the social roots of biological processes: as long as a simple model explains a trend, such as the slight population decline in 1934, there does not seem to be any need to suppose that a more complex model or exogenous causes need to be considered. Thus, as a contributing factor to the historical narrative about the Gran Paradiso, Jacobson et al.’s (2004) model acts as an alarm bell, signaling from which point onwards other causes beside climate and population density also need to be considered. It is obvious that there will always be other addictive factors at play: what the model is able to do is to indicate where such factors are just background noise that can be cancelled out by the main causal ecological feedback processes and from which point onwards other, more nuanced approaches become necessary. This is true for both the 1930s, when, because of the lack of additional quantitative data, a narrative approach has to preferred, and the late 1990s, for which new models are still being researched that may better explain the major trend shifts.

## Footnotes

1 I thank Jost Graf von Hardenberg, Institute of Atmospheric and Climate Sciences (ISAC-CNR) in Turin, for kindly performing the necessary modeling and calculations using MatLab. Obviously, as author, I take full responsibility for any errors or misinterpretations.

2 Translation by the author.

3 The historical snow data have been extracted from the *Bollettino Mensile* and the *Annali Idrologici* of the Ufficio Idrografico del Po. The data from 1917 to 1927 and from 1936 to 1945 were collected by the Parma office; those from 1928 to 1935 by the Turin office. Full texts of all the Ufficio Idrografico del Po publications can be accessed on the ISPRA website (“Consultazione annali idrologici” n.d.). The precipitation data for Gran Paradiso used throughout this paper are courtesy of ARPA, the regional environmental agency of Piedmont, via Jost Graf von Hardenberg (Hardenberg to author 2009).

4 The ibex population data for the Swiss National Park were kindly provided by the park’s management (Filli to author 2011), while MeteoSwiss provided precipitation data for the Buffalora station near the national park (“IDAWEB” n.d.).
